# From counting to clustering: AI-defined “diffuse” vs. “focal” spatial patterns as the new precision biomarker for triple-negative breast cancer

**DOI:** 10.3389/fimmu.2026.1796137

**Published:** 2026-03-11

**Authors:** Liyun Cai, Pengping Li

**Affiliations:** 1 Lou Ta Town Community Health Service Center, Xiaoshan District, Hangzhou, China; 2 The First People’s Hospital of Xiaoshan District Hangzhou, The Xiaoshan Affiliated Hospital of Wenzhou Medical University, Zhejiang, China

**Keywords:** artificial intelligence, precision biomarker, spatial clustering, triple-negative breast cancer, tumor-infiltrating lymphocytes

## Introduction

The integration of immunotherapy into the neoadjuvant treatment has changed the clinical management of triple-negative breast cancer (TNBC). And, the “one-size-fits-all” approach to patient selection is gradually being replaced by individualized precision treatment. Although Nature Medicine forecasts for 2025 rightly emphasized the expansion of precision trials (1), clinical practice still relies on quantifying the abundance of tumor-infiltrating lymphocytes (TILs) or PD-L1 expression (or Combined Positive Score, CPS), basing on current evidence-based basis. We write to highlight a pivotal shift emerging from late 2025 literature: the transition from “counting” immune cells to decoding their spatial clustering patterns.

A multicenter study published in December 2025 has provided an interesting and surprising phenomenon that the spatial architecture of TILs dictates therapeutic response in TNBC, independent of total cell count (2). Utilizing an artificial intelligence (AI)-based spatial clustering classifier, the investigators identified two distinct immune landscapes: a “Diffuse Immune Subtype” and a “Focal Hotspot Subtype”.

## Spatial heterogeneity as a determinant of response

To our knowledge, a tumor with high TIL density is usually considered as “immune-hot” subtype, with higher immunotherapy response rate as comparing to the lower one. However, this new AI-driven analysis shows a different scope. Patients with the “Focal Hotspot” subtype, which characterized by dense but isolated clusters of immune cells, often exhibit poorer pathological complete response (pCR) rates compared to those with “Diffuse” infiltration, where immune cells are spread throughout the tumor stroma (2). This phenomenon explains a persistent clinical paradox: why some patients with seemingly “high-TIL” tumors fail to respond to immune checkpoint inhibitors (ICIs). This research suggests that “Focal” patterns may represent a localized or limited immune response, which is the potential result from tumor fibrosis or tertiary lymphoid structure dysregulation, while “Diffuse” patterns indicate a pervasive anti-tumor effect.

## Clinical implications for 2026

The urgency of this shift was also mentioned in discussions at SABCS 2025 regarding the APHINITY trial analysis (3). Although the SABCS data focused on HER2-positive disease, the conference consensus underscored a universal truth: manual scoring is reaching its limit. So, as a novel standpoint or future transformation, the integrating the AI-defined “Diffuse” vs. “Focal” metrics is no longer a concept but a tangible necessity to refine clinical workflows for TNBC:

1. Refining Prognosis: The “Diffuse” signature serves as an independent predictor of prolonged disease-free survival (DFS), offering a more granular risk stratification than current AJCC staging (2).
2. Stratifying HER2-Low TNBC: The study further elucidated that the “Diffuse” pattern correlates with better outcomes specifically in the HER2-low TNBC subgroup, a population that is increasingly becoming a distinct therapeutic target (2).

## Conclusion

The era of merely quantifying “how many” immune cells are present is ending. The objective data from late 2025 compels us to ask “how are they arranged?”. The integration of AI-powered spatial clustering—specifically the differentiation of Diffuse vs. Focal phenotypes—into future clinical trials is essential for refining patient stratification and improving the predictive accuracy of treatment outcomes. This spatial logic is the missing link needed to close the gap between biomarker positivity and actual patient survival.

## References

[1] Webster P Healey N . Eleven clinical trials that will shape medicine in 2025. Nat Med. (2024) 30:3384–8. doi: 10.1038/s41591-024-03383-y, PMID: 39668215

[2] Xie T Ai S Liu C Luo B Yan H Zhang H . Focal hotspot and diffuse immune subtypes of tumor-infiltrating lymphocytes: AI-powered spatial clustering classification and its clinical relevance to HER2 expression in triple-negative breast cancer. J Transl Med. (2025) 24(1):111. doi: 10.1186/s12967-025-07608-7, PMID: 41437085 PMC12836918

[3] Salgado R Gonzalez LEL Giudici F Rojo F Comerma L Wienert S . Abstract GS1-05: Prognostic and predictive associations of manual, digital and AI-derived tumor infiltrating lymphocytes-scoring: A retrospective analysis from the Phase III APHINITY trial. Clin Cancer Res. (2026) 32:GS1-05-GS01-05.

